# Rapid change of fecal microbiome and disappearance of *Clostridium difficile* in a colonized infant after transition from breast milk to cow milk

**DOI:** 10.1186/s40168-016-0198-6

**Published:** 2016-10-07

**Authors:** Manli Y. Davis, Husen Zhang, Lera E. Brannan, Robert J. Carman, James H. Boone

**Affiliations:** 1 TechLab, Inc., 2001 Kraft Drive, Blacksburg, VA 24060 USA; 2 Virginia Polytechnic Institute and State University, Blacksburg, VA 24061 USA

**Keywords:** *C. difficile*, Infant gut microbiome, Human milk

## Abstract

**Background:**

*Clostridium difficile* is the most common known cause of antibiotic-associated diarrhea. Upon the disturbance of gut microbiota by antibiotics, *C. difficile* establishes growth and releases toxins A and B, which cause tissue damage in the host. The symptoms of *C. difficile* infection disease range from mild diarrhea to pseudomembranous colitis and toxic megacolon. Interestingly, 10–50 % of infants are asymptomatic carriers of *C. difficile.* This longitudinal study of the *C. difficile* colonization in an infant revealed the dynamics of *C. difficile* presence in gut microbiota.

**Methods:**

Fifty fecal samples, collected weekly between 5.5 and 17 months of age from a female infant who was an asymptomatic carrier of *C. difficile*, were analyzed by 16S rRNA gene sequencing.

**Results:**

Colonization switching between toxigenic and non-toxigenic *C. difficile* strains as well as more than 100,000-fold fluctuations of *C. difficile* counts were observed. *C. difficile* toxins were detected during the testing period in some infant stool samples, but the infant never had diarrhea. Although fecal microbiota was stable during breast feeding, a dramatic and permanent change of microbiota composition was observed within 5 days of the transition from human milk to cow milk. A rapid decline and eventual disappearance of *C. difficile* coincided with weaning at 12.5 months. An increase in the relative abundance of *Bacteroides* spp., *Blautia* spp*.*, *Parabacteroides* spp., *Coprococcus* spp., *Ruminococcus* spp., and *Oscillospira* spp. and a decrease of *Bifidobacterium* spp., *Lactobacillus* spp., *Escherichia* spp., and *Clostridium* spp. were observed during weaning. The change in microbiome composition was accompanied by a gradual increase of fecal pH from 5.5 to 7.

**Conclusions:**

The bacterial groups that are less abundant in early infancy, and that increase in relative abundance after weaning, likely are responsible for the expulsion of *C. difficile.*

**Electronic supplementary material:**

The online version of this article (doi:10.1186/s40168-016-0198-6) contains supplementary material, which is available to authorized users.

## Background

*Clostridium difficile* (*C. difficile*), a gram-positive spore-forming anaerobic bacterium, accounts for half a million cases of diarrhea [1] and 14,000 deaths [2] annually in the USA. The symptoms of *C. difficile* disease in adults range from mild diarrhea to pseudomembranous colitis and toxic megacolon. Up to 50 % of infants are asymptomatic carriers of *C. difficile* [3–5]. The percentage of infants colonized is higher at the beginning of life, an average of 37 % at 1 month of age [6], and declines to 30 % between 1 and 6 months of age. At the end of the first year, the colonization rate drops to 10 % [6]. The cause of this decrease in colonization is unknown, and most studies have reported data through these events as an aggregate of many individuals, unlinked to specific events in each participant during that timeframe. *C. difficile* disease results from tissue damage caused by two toxins, A (TcdA) and B (TcdB), which are produced by toxigenic *C. difficile* strains. Surprisingly, toxin concentration in asymptomatic infants can be similar to the level in adults with pseudomembranous colitis [7].

*C. difficile* infection (CDI) is associated with a disturbance in gut microbiota. Antibiotic exposure is the most important risk factor for CDI. The usage of broad-spectrum antibiotics, such as clindamycin, aminopenicillins, cephalosporins, and fluoroquinolones disturbs the normal gut microbiota and predisposes persons to subsequent CDI [8–10]. Restoration of the disturbed microbiota by bacteriotherapy is effective in treating recurrent CDI [11].

The high *C. difficile* colonization rate in infants may be contributed to the fact that the commensal microbiota in pre-weaned infants dominated by *Bifidobacterium* spp. and *Lactobacillus* spp. [12] might be more permissive to the colonization of *C. difficile* than adult microbiota dominated by *Bacteroidetes* spp. and *Firmicutes* spp. [13]. The gut microbiome structure can be rapidly altered by changes in diet [14]. Thus, the notable differences in diet likely contribute to the difference in the microbiota composition of infant and adult gut. The most significant change of diet in infancy is weaning. Weaning is the process of introducing an infant to an adult diet and withdrawing the supply of mother’s milk. In this study, weaning refers to the transition from human milk to cow milk with the same supplemental solid food intake. We observed in this study that weaning was associated with the maturation of infantile gut microbiota to adult-like gut microbiota.

The aim of this study was to evaluate *C. difficile* colonization in an infant pre- and post-weaning. Solid food was introduced at the age of 4 months with the continued feeding of human milk before stool collection began. The major change in the infant’s diet was the cessation of breast milk and introduction to cow milk at a single day around the age of 12 months. Fecal samples were collected from an infant weekly from 5.5 months of age to 17 months of age. The infant was an asymptomatic carrier at the beginning of the study and transitioned to *C. difficile* negative during the testing period. The composition of infant fecal microbiota was analyzed retrospectively to investigate the cause of the disappearance of *C. difficile*. We hypothesize that breast milk promotes infant-like gut microbiota which allows the colonization of *C. difficile*.

## Methods

### Infant and sample collection

A female infant was identified as an asymptomatic carrier of *C. difficile* at age 5.5 month using a combination of immunoassay detection of glutamate dehydrogenase (GDH) and bacterial culture. The infant stool sample was tested positive for GDH on *C. DIFF QUIK CHEK COMPLETE®* (TechLab, Inc, Blacksburg, VA), and *C. difficile* colonies were isolated from infant stool samples using ethanol-shock spore enrichment method [15]. The infant was delivered through Cesarean delivery and fed exclusively with breast milk until the age of 4 months when solid food was gradually introduced. Solid food included oatmeal, fruits, yogurt, and protein such as tofu, eggs, and meat. The composition of solid food remained constant throughout the duration of sample collection. An average of 20 oz. of human milk or cow milk was consumed daily throughout this study. Formula was never given to the infant. Fecal samples were collected weekly for 50 weeks starting on 1 Nov. 2013 when the infant was 5.5 months of age. Samples were collected at home and stored at 2–8 °C overnight before they were aliquoted and stored frozen at −20 °C until analyzed. The study was approved by the TechLab Institutional Review Board and included informed consent obtained from the mother.

### Measurement of pH of fecal samples

Fecal samples stored at −20 °C were thawed, brought to room temperature, and homogenized. The pH of fecal samples was measured by placing Micro Combination pH Electrode (Thermo Scientific, Waltham, MA) directly into the center of the homogenized samples at room temperature.

### *C. difficile* isolation and ribotyping

*C. difficile* isolation was done using ethanol-shock spore enrichment method [15]. Tenfold serial dilutions were made before plating on to cycloserine-cefoxitin-fructose agar (CCFA) plates for spore count. Isolated colonies were used to inoculate prereduced, anaerobically sterilized brain heart infusion broth (Anaerobe Systems, Morgan Hill, CA) and the inoculated media was incubated at 37 °C for 72 hours anaerobically. Genomic DNA was isolated from *C. difficile* colonies and PCR ribotype was determined using the procedure developed by Stubbs et al. [16].

### *C. difficile* antigen and toxin quantification

The amount of GDH, quantified to nanogram over gram on an ELISA (*C. DIFF CHEK®* - 60, TechLab, Inc, Blacksburg, VA) using a standard curve generated with recombinant GDH, was used to indicate the presence of metabolically active vegetative *C. difficile* in fecal samples. The presence or absence of toxin A and B was determined by *C. DIFF QUIK CHEK COMPLETE®* (TechLab, Inc, Blacksburg, VA). Toxin B in the fecal samples was confirmed by the *TOX-B TEST* (TechLab, Inc, Blacksburg, VA). Toxin B-specific cytotoxicity was confirmed by the neutralization of fecal samples using toxin B-specific antibodies on human foreskin cells (Diagnostic Hybrids, Athens, OH). Briefly, fecal supernatant fluids were prepared from specimens diluted 1:10 and clarified by centrifugation and filtration. The cytotoxicity neutralization with specific antitoxin was recorded 48 h post-inoculation. Fecal samples that tested positive on the *TOX-B TEST* were diluted tenfold and re-tested for further titration.

### Sample preparation for 16S rRNA gene analysis

Genomic DNA was isolated from 50 stool samples with PowerSoil DNA Isolation Kit (MoBio), following the manufacturer’s instructions. The V4 region of 16S rRNA gene was amplified with forward primer 515F and GoLay barcoded 806R reverse primers [17]. Duplicate PCRs were set up using different reverse primers to verify the reproducibility of the amplification. The resulting 100 amplicons were purified with QIAquick PCR Purification Kit (Qiagen, Hilden, Germany) and pooled and sequenced by paired-end of 150 cycles on a MiSeq sequencer (Illumina, San Diego, CA, USA). The average results of the two technical duplicates were used in the subsequent analyses.

### Taxonomy assignments and community structure analyses

The sequencing results were processed using Quantitative Insights Into Microbial Ecology (QIIME) [18]. The bi-directional reads were joined using QIIME’s default join_paired_ends script. The merged reads were filtered based on Phred quality score ≥20, which corresponds to sequencing error rate ≤0.01. Chimeras were identified using USEARCH61 [19] and removed from downstream analysis. After the removal of chimeras, the remaining sequences were mapped into operational taxonomic units (OTUs) against the Greengenes reference sequences (version 2013.5) with the program UCLUST [19]. Microbial taxonomy was assigned using a naïve Bayesian classifier trained with the Greengenes 2013.5 reference sequences [20, 21]. The level of species was classified by running BLAST [22] against the Greengenes data set. OTUs which showed significant change in relative abundance was identified using QIIME’s “group_significance.py” script with Kruskal-Wallis test. Principle coordinates were calculated using unweighted UniFrac metrics [23]. QIIME commands used to generate the principal coordinate plots were included in Additional file 1.

## Results

### Fluctuation of *C. difficile* colony count and ribotype switch in a colonized infant

**Fig. 1:**
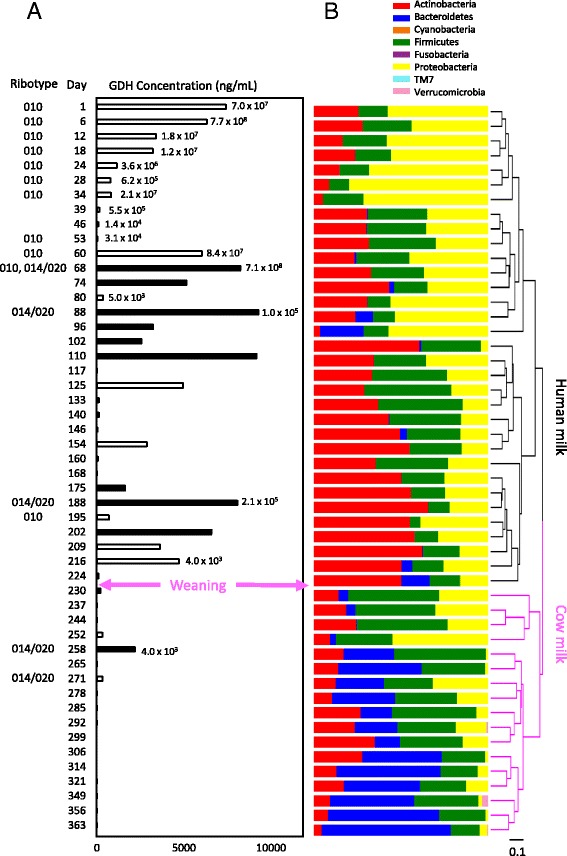
The dynamics of *C. difficile* colonization and microbiome composition in infant samples. **a** The amount of GDH quantified by ELISA was used to indicate the relative abundance of *C. difficile* in infant fecal samples. Samples that tested positive for *C. difficile* toxins are indicated by *black bars* while samples that tested negative for toxins are indicated by *open bars. C. difficile* spore ribotype and spore count are presented for a subset of samples. The switch from mother’s milk to cow milk was indicated with the *purple arrowed lines*. Sample collection started on day 1 when the infant was 5.5 months old. **b** The microbiome composition of infant fecal samples are profiled at the phylum level. The microbiota samples are grouped by hierarchical clustering based on UniFrac distances. Samples collected before weaning and samples collected after weaning formed two distinct clusters

The transition from human milk to cow milk paralleled with rapid disappearance of *C. difficile* in this study. The infant was initially culture positive for *C. difficile* and remained positive for a period of 292 days. GDH, a metabolic enzyme produced by vegetative *C. difficile*, was quantified and used as a marker to indicate the active growth of vegetative *C. difficile*. Among the samples that were culture positive for *C. difficile*, a variation of GDH level between less than 1 ng to over 9000 ng GDH per gram of feces was observed (Fig. 1a). The number of *C. difficile* spores in feces was also performed on a subset of the samples to check the bacterial load. The lowest spore count for *C. difficile* positive stools was 4.0 × 10^3^/g in samples collected on days 216 and 258 of the testing period. The highest spore count was 7 × 10^8^/g in the samples collected on day 6 and 68. Based on spore counts, we observed a more than 100,000-fold decrease in *C. difficile* load between day 68 and days 80 and 216. Note that although the amount of GDH reflects the amount of vegetative cells present in samples, the amount of GDH does not strictly correlate with the spore count of *C. difficile* from the same fecal sample. No detectable amount of GDH was present in samples collected on and after day 278 to the end of the testing period, confirming along with the absence of cultivable *C. difficile*, the disappearance of *C. difficile*.

Eleven out of 50 stool samples were positive for *C. difficile* cytotoxicity over a 191-day period. In Fig. 1a, samples that tested positive for toxins are indicated with solid black bars, whereas samples that were negative for toxins are represented with open bars. Toxigenic *C. difficile* was first detected on day 68. A switching between toxin negative samples and toxin positive samples was observed throughout 2/3 of the testing period. Interestingly, based on toxin titration in cytotoxicity assays, samples collected on days 68, 74, and 96 contained 10 times higher amounts of toxin than amounts shown to cause severe CDI in adults, yet the infant remained asymptomatic.

Ribotyping was performed periodically to identify the ribotypes of the *C. difficile* isolated from a subset of stool samples (Fig. 1a). For the first 60 days of the testing period, non-toxigenic ribotype 010 was present. In the sample collected on day 68, the first toxin positive sample in the series, both non-toxigenic ribotype 010 and toxigenic ribotype 014/020 were detected. Ribotype 014/020 was also identified in toxin positive samples collected on days 88, 188, 258, and 271. The sample collected on day 271 contained no detectable amount of toxin by cytotoxicity assay and showed a low GDH level. Although 014/020 has been reported to be the most commonly identified ribotype of *C. difficile* isolated from diarrheic pediatric CDI cases [24], all stool samples containing 014/020 in this study were solid and the infant remained asymptomatic.

### Rapid change in microbiota composition at phylum level after weaning

**Fig. 2:**
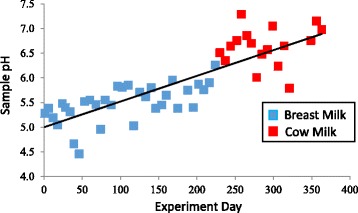
Gradual pH increase in infant fecal samples. The pH of infant fecal samples collected weekly from 5.5 months of age (day 1) to 17 months of age (day 363) were tested. Weaning occurred on day 224. The samples collected before weaning are represented by *blue squares* while samples collected after weaning are represented by *red squares*. A gradual pH increase was observed throughout the test period

Diet greatly influence gut microbiome [14] by providing various substrates that promote the growth of different groups of bacteria. At the beginning of the study, the infant was 5.5 months of age with solid food already introduced. The pH of the fecal samples was tested. Breastfed infants produce stool with pH around 5, which was also observed at the beginning of this study (Fig. 2). The fecal pH gradually increased to 7 toward the end of the sample collection. Coincide with the increase of pH of infant fecal samples, a gradual increase of microbial diversity represented by Shannon diversity index [18] was observed throughout this study (Additional file 2: Figure S1).

**Fig. 3:**
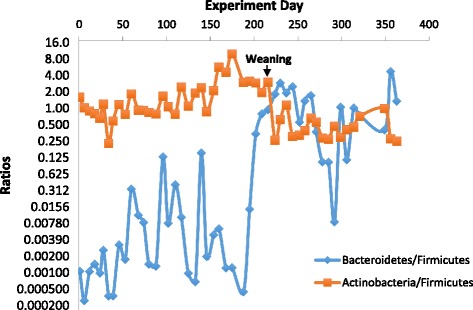
Weaning changes the ratio of bacteria phyla in the infant gut. When the infant was weaned off breast milk to cow milk, the *Bacteroidetes* to *Firmicutes* ratio increased dramatically and the *Actinobacteria* to *Firmicutes* ratio decreased slightly. Weaning happened between sample points day 224 and 230

To investigate the exclusion of *C. difficile* from the colonized infant, microbial structure was analyzed by 16S rRNA gene sequencing of the total microbial community. Taxonomy assignments and community structure analyses were done as described [25]. In Fig. 1b, the structure of the fecal microbiota is displayed and color-coded by the various bacterial phyla that were present. A striking decrease of *Actinobacteria* was observed between samples collected on days 224 and 230. The record of diet revealed the complete termination of human milk feeding on day 225. Further analysis of the ratio of dominant bacterial phyla indicated that within 5 days from the cessation of human milk and its replacement by cow milk, the *Bacteroidetes* to *Firmicutes* ratio increased dramatically while the *Actinobacteria* to *Firmicutes* ratio decreased slightly (Fig. 3). These changes in the microbiome structure were sustained for 134 days till the end of the testing period. The *Bacteroidetes* to *Firmicutes* ratio started to increase on days 216 and 224, just before the cessation of human milk. On these days, small amounts (approximately 4 oz) of cow milk were given to the infant in addition to human milk. Whether addition of cow milk initiated these changes in the microbiome remains to be determined.

**Fig. 4:**
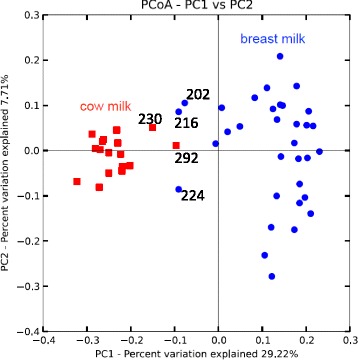
The structure of microbial communities is distinct before and after weaning. Principal coordinate analysis based on unweighted UniFrac metrics indicates that microbiota community structures are distinct between pre-weaning (*blue dots*) and post-weaning (*red squares*) fecal samples. Weaning happened between experiment days 224 and 230. On experiment day 292 when the infant produced two loose stools, significant alteration of the microbiota composition was observed

The rapid change in microbial structure that occurred within 5 days of weaning was also indicated in a principal coordinate analysis based on unweighted UniFrac metrics (Fig. 4). Microbial community structures were distributed into two distinct clusters that represent pre-weaning and post-weaning fecal samples. Data representing days 224 and 230 were both in the positions connecting the two communities of data points, indicating that a major transition of microbial structure occurred simultaneously with weaning.

### Microbial structure change at genus and species level coincided with weaning

**Fig. 5:**
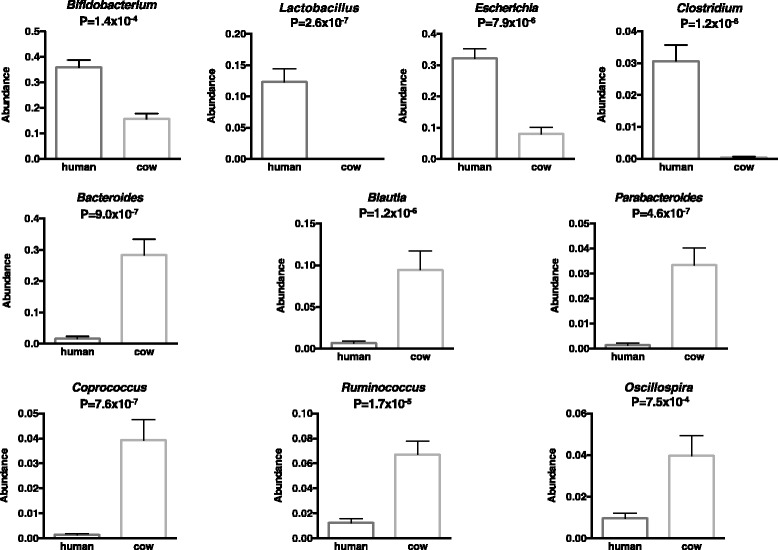
Genera of bacteria which changed significantly before and after weaning. The comparison of microbiota composition pre- and post-weaning was done using parametric *t* test (*p* values <0.005). The relative abundance of bacteria genera was calculated as percentage of the total bacteria detected. The relative abundance of *Bifidobacterium* spp., *Lactobacillus* spp.*, Escherichia* spp., and *Clostridium* spp. decreased post-weaning while the relative abundance of *Bacteroides* spp., *Blautia* spp., *Parabacteroides* spp., *Coprococcus* spp., *Ruminococcus* spp., and *Oscillospira* spp. increased

A second comparison of microbiota composition, with data gathered into pre-weaning (days 1–224) and post-weaning (days 225–363) groups, was done using a parametric *t* test (*p* values <0.005). At the genus level, the relative abundance of *Bacteroides* spp., *Blautia* spp., *Parabacteroides* spp., *Coprococcus* spp., *Ruminococcus* spp., and *Oscillospira* spp. dramatically increased post-weaning while the relative abundance of *Bifidobacterium* spp., *Lactobacillus* spp., *Escherichia* spp., and *Clostridium* spp. decreased (Fig. 5). The phylogenetic designation of the genera which changed significantly during weaning are summarized in Additional file 3: Table S1.

In addition, 69 operational taxonomic units (OTUs) showed significant change in relative abundance pre- and post- weaning (FDR *p* value ≤0.01). The majority of these OTUs were assigned to species that have not been isolated in culture. Among the cultivable species, *Bifidobacterium adolescentis*, *Bifidobacterium bifidum*, *Escherichia coli*, and *Lactobacillus zeae* were more abundant before weaning, whereas *Bacteroides caccae*, *Bacteroides uniformis*, *Corynebacterium durum*, *Ruminococcus callidus*, *Ruminococcus gnavus*, and *Ruminococcus torques* were more abundant after weaning (Table 1).

**Table 1:** Weaning led to a change in relative abundance of 10 cultivable bacteria species

Species name	Representing OTUs	FDR P value	Change in relative abundance after weaning
*Bacteroides caccae*	5	1.55 × 10^−7^	Increase
*Bacteroides uniformis*	3	1.08 × 10^−4^	Increase
	205	1.07 × 10^−5^	
	326	2.91 × 10^−5^	
*Corynebacterium durum*	109	1.43 × 10^−3^	Increase
*Ruminococcus callidus*	65	7.13 × 10^−3^	Increase
*Ruminococcus gnavus*	6	5.09 × 10^−5^	Increase
	217	3.50 × 10^−5^	
	249	2.30 × 10^−6^	
	322	2.83 × 10^−3^	
*Ruminococcus torques*	28	4.96 × 10^−7^	Increase
*Bifidobacterium adolescentis*	128	3.57 × 10^−5^	Decrease
	180	2.39 × 10^−3^	
	233	2.05 × 10^−3^	
	329	5.23 × 10^−7^	
*Bifidobacterium bifidum*	162	6.61 × 10^−4^	Decrease
	267	2.55 × 10^−4^	
*Escherichia coli*	1	3.50 × 10^−5^	Decrease
	121	3.06 × 10^−3^	
	131	1.73 × 10^−3^	
	144	1.83 × 10^−4^	
	189	7.93 × 10^−3^	
	223	2.47 × 10^−3^	
	243	7.41 × 10^−4^	
*Lactobacillus zeae*	4	2.66 × 10^−3^	Decrease
	169	3.21 × 10^−3^	
	247	2.83 × 10^−3^	

## Discussion

The primary goal of this study was to investigate the dynamics of *C. difficile* colonization in an infant before and after the transition from human milk to cow milk. The high rate of asymptomatic carriage of *C. difficile* in infants is well documented but until now the transition from carrier state to non-carrier state has not been characterized. We conducted a longitudinal study on an infant to investigate the transition from *C. difficile* colonization to a *C. difficile*-negative state. When sample collection of this study started the infant was colonized with 7.0 × 10^7^ CFU/g of *C. difficile* spores per gram of feces at 5.5 months of age. When the study ended, the infant was 17 months old and was free of *C. difficile*. As high as 7.1 × 10^8^ CFU/g of toxigenic *C. difficile* was detected in a sample collected on day 68. This bacterial load was high enough to cause CDI in adults [26] but did not cause diarrhea in this infant. The amount of GDH, which is produced by vegetative *C. difficile*, was quantified on an ELISA as an indicator of vegetative growth. Close to 10,000 ng/g feces of GDH was detected in the stool collected on experiment day 88. Less than 20 ng/g feces of GDH concentration was detected in the last 10 samples (days 278 to 363), confirming the diminished growth of *C. difficile*. This is the first study to use the combination of GDH concentration and spore count to investigate the dynamics of both vegetative and spore forms of *C. difficile*, respectively, in a colonized infant. The presence of GDH was shown to be both a sensitive and specific biomarker of *C. difficile* [27]. The high spore count of *C. difficile* in the infant stool makes infants reservoirs of *C. difficile*.

Ribotype switches between non-toxigenic and toxigenic *C. difficile* were captured in this longitudinal study. At the beginning of the testing period, the infant was colonized with non-toxigenic *C. difficile* ribotype 010. On day 68, a mixture of non-toxigenic ribotype 010 and toxigenic ribotype 014/020 coexisted. On day 88, only toxigenic ribotype 014/020 was isolated (Fig. 1a). The acquisition of two different strains of *C. difficile* indicates that *C. difficile* can be acquired from the environment, and the colonization with different strains of *C. difficile* represented transient events. Although the *C. difficile* spore counts and GDH amounts varied, colonization with *C. difficile* was sustained throughout the human milk-fed stage from day 1 to 224.

Samples collected on days 68, 74, 88, and 96 contained 10 times the amount of *C. difficile* toxin that can cause *C. difficile* disease in adults, yet the infant remained asymptomatic on these days. This is consistent with the report from Viscidi et al. that the concentrations of toxins in the stool of healthy infants can reach the levels of toxins that cause severe disease in adults [7]. This study is one of the first to document the extreme temporal variability of both vegetative cells and spores that can be present in an asymptomatic infant. The ineffectiveness of toxins on infants was hypothesized to be due to the lack of receptor on infant gut epithelial cells [28] but such receptor has not been identified.

A second goal of this study was to document the detailed longitudinal changes in gut microbiota during the first year and a half of life of an infant. The maturation of infant microbiota is thought to be a gradual process until reaching the composition of adult microbiota at the age of 3 years [29]. However, in this unique case of an infant experiencing abrupt termination of human milk feeding and transition to cow milk feeding, the infant microbiota shifted from *Bifidobacterium* spp. and *Lactobacillus* spp. dominated microbiota to *Bacteroidetes* spp. and *Firmicutes* spp. dominated microbiota within 5 days. It appears that cessation of breastfeeding rather than introduction of solid foods caused the shift from *Bifidobacterium* spp. and *Lactobacillus* spp. dominated microbiota to *Bacteroidetes* spp. and *Firmicutes* spp. dominated microbiota, as solid foods had been introduced 6 weeks before the start of the study and continued to be part of the infant’s diet. This agrees with the results of Backhed et al. [12]. There are significant differences between human milk and cow milk. Human milk contains less than half the protein, about 50 % more lactose [30] and a significantly higher amount of oligosaccharides [31, 32]. The oligosaccharides in human milk are resistant to digestion in the small intestines and promote the growth of *Bifidobacteria* once they reach the colon [33, 34]. The differences in composition of human milk and cow milk produce environments that favor different groups of bacteria pre- and post-weaning. The timing of microbiota maturation may then be more dependent on the timing of weaning than is currently recognized. For example, the sudden decrease of oligosaccharides after the cessation of human milk feeding may explain the rapid decrease of *Bifidobacteria* after weaning observed in this study.

The disappearance of *C. difficile* after weaning also coincided with the shift from *Bifidobacterium* spp. and *Lactobacillus* spp. dominated microbiota to *Bacteroidetes* spp. and *Firmicutes* spp. dominated microbiota. *C. difficile* does not carry the genes to synthesize hydrolytic enzymes capable of cleaving monosaccharides from oligosaccharide side chains [35] and must depend upon acquisition of sugars from other members of the colonic microbiome. *Bifidobacterium bifidum*, which is abundant in infant microbiota, is capable of degrading mucin *O*-glycans [36]. Free sialic acid, the mucin degradation product made by *Bifidobacterium bifidum*, serves as an energy source of *C. difficile* [37]. Infantile gut microbiota likely accommodate *C. difficile* by producing metabolic substrates such as free sialic acid*.* A significant decrease of *Bifidobacterium bifidum* was observed after weaning in this study. The relative abundance of *C. difficile* positively correlated with the relative abundance of OTUs 162 and 267 representing *C. bifidum* with coefficients 0.37 and 0.29 and *p* values 0.007 and 0.04, respectively. The decreased relative abundance of free sialic acid-producing bacterial species restricts the energy source of *C. difficile* and may contribute to the disappearance of *C. difficile*.

In addition, adult gut microbiota form a barrier against *C. difficile* colonization by competing for metabolic substrates and producing inhibitors against *C. difficile* [38]. For example, an inverse association between *C. difficile* and the relative abundance of members of the *Bacteroidetes* phylum and of other *Clostridium* spp. in human intestines has been observed [39]. *Bacteroides* spp. have been shown to form a resistance barrier against *C. difficile* by competing for monomeric sugars such as glucose, *N*-acetylglucosamine, succinate, and sialic acids [35, 40–43]. The percentage of *Bacteroides* spp. increased from 0.02 to 48 % in the gut microbiota from the first sample to the last sample in our study. In particular, *Bacteroides caccae* and *Bacteroides uniformis* were more abundant post-weaning. The increase of *Bacteroides* spp. after weaning limited the carbon source of *C. difficile* and therefore contributed to the disappearance of *C. difficile*.

The barrier against *C. difficile* colonization in *Bacteroidetes* spp. and *Firmicutes* spp. dominated microbiota also results from metabolic products produced by the adult-like microbiota that inhibit the germination and growth of *C. difficile*. For example, bile acids strongly influence the germination of *C. difficile* spores and the growth of vegetative *C. difficile* cells. Primary bile acids stimulate *C. difficile* spore germination [44] whereas secondary bile acids inhibit the growth of vegetative *C. difficile* [45, 46]. Importantly, the dynamic balance of bile acid composition in the colon is influenced by the commensal bacterial species in the gut. Primary bile acids are deconjugated by bacteria, such as *Clostridium scindens*, and further modified into secondary bile acids which are inhibitory to *C. difficile* in the colon [47]. *Ruminococcus gnavus*, one of the cultivable bacteria species identified in this study that increased in relative abundance after weaning, converts lithocholate to ursodeoxycholate and plays a major role in ursodeoxycholate formation in the colon [48]. Ursodeoxycholate inhibits *C. difficile* spore germination [45]. The increased level of *R. gnavus* after weaning may further contribute to the disappearance of *C. difficile* by increasing the concentration of ursodeoxycholate in the colon.

Previous studies have indicated that *Ruminococcus callidus* is part of the resistance barrier against *C. difficile. R. callidus* was found to be more abundant in healthy people than in Crohn’s disease patients, who are also at higher risk of CDI [49, 50]. *R. callidus* is associated with the gut mucosa and is restored after fecal transplants [51]. In our study, the increased relative abundance of *R. callidus* after weaning may contribute to the disappearance of *C. difficile*.

Whether children under the age of 2 should be tested and/or treated for CDI is debatable. Rates of positive *C. difficile* tests were the same among children with or without diarrhea [52]. More than 20 % of children with diarrhea who tested positive for *C. difficile* also tested positive for other pathogens, making it difficult to determine whether *C. difficile* was the true cause of diarrhea or part of the commensal microbiota [53]. Even when children under 2 years of age were diagnosed with CDI, the outcomes of treated and untreated CDI were not significantly different [54]. The guidelines published by the National Health Services (NHS) in Britain suggested that children under the age of 2 not be tested for CDI [55]. In this study, the toxin was not detectable by cytotoxicity assay in the sample collected on day 271, but *C. difficile* 014/020 could be grown on CCFA plates and its DNA ribotyped. Thus, although present, the 014/020 *C. difficile* was not producing toxin. These results demonstrate once again that even when toxigenic *C. difficile* can be detected by molecular means, it may not be causing disease. Molecular assays targeting genes encoding the *C. difficile* toxins will, in such cases, produce false positive results. Whether toxigenic *C. difficile* causes disease or not appears to depend on the age-related susceptibility of children and the composition of the gut microbiota. Better understanding of the colonization of *C. difficile* in children will lead to accurate diagnosis and proper treatment of CDI in children.

## Conclusions

This longitudinal study of *C. difficile* colonization in an infant revealed up to 10^5^-fold fluctuation of *C. difficile* count, colonization with both non-toxigenic and toxigenic *C. difficile* strains, as well as rapid shift from *Bifidobacterium* spp. and *Lactobacillus* spp. dominated microbiota to *Bacteroidetes* spp. and *Firmicutes* spp. dominated microbiota within 5 days of weaning from human milk. The disappearance of *C. difficile* after weaning likely is due to a resistance barrier formed by the adult-like microbiota. Bacteria species identified in this study that are more abundant in adult-like post-weaning microbiota may be components of the resistant barrier against CDI.

## Additional files

Additional file 1: QIIME commands used to generate the principal coordinate plots. (TXT 2 kb)
Additional file 2: Figure S1. Shannon index of the infant microbiota increases with time. (PDF 5 kb)
Additional file 3: Table S1. Bacteria genera changed significantly after weaning. (XLSX 9 kb)

## Abbreviations

CCFA: Cycloserine-cefoxitin-fructose agar
CDI: *C. difficile* infection
GDH: Glutamate dehydrogenase
NHS: National Health Services
OTUs: Operational taxonomic units
QIIME: Quantitative Insights Into Microbial Ecology
TcdA: *Clostridium difficile* toxin A
TcdB: *Clostridium difficile* toxin B
