# Supplementary figures and images for: Rapid change of fecal microbiome and disappearance of Clostridium difficile in a colonized infant after transition from breast milk to cow milk
Source: Microbiome. 2016 Oct 7;4:53. doi: 10.1186/s40168-016-0198-6 (PMC5055705; doi:10.1186/s40168-016-0198-6)

R-squared = 0.39  
p-value:  $6.8e-07$

Shannon Diversity

0

100

200

300

Days

1.5

2.0

2.5

3.0

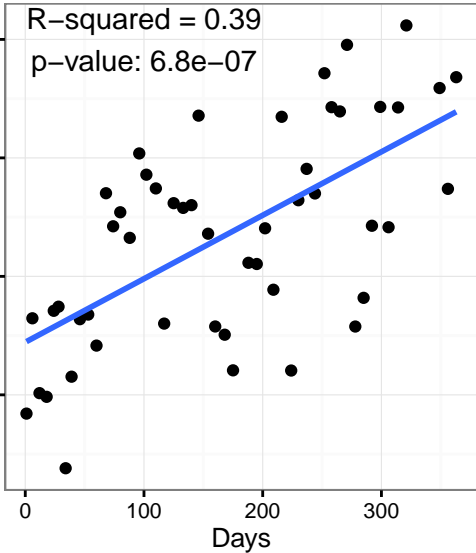

Supplement: Additional file 2: Figure S1. — Shannon index of the infant microbiota increases with time. (PDF 5 kb) [file 40168_2016_198_MOESM2_ESM.pdf]
